# Hygroregulation, a key ability for eusocial insects: Native Western European honeybees as a case study

**DOI:** 10.1371/journal.pone.0200048

**Published:** 2019-02-08

**Authors:** Iris Eouzan, Lionel Garnery, M. Alice Pinto, Damien Delalande, Cátia J. Neves, Francis Fabre, Jérôme Lesobre, Sylvie Houte, Andone Estonba, Iratxe Montes, Télesphore Sime-Ngando, David G. Biron

**Affiliations:** 1 Laboratoire Microorganismes: Génome et Environnement, UMR CNRS 6023, Université Clermont-Auvergne, Campus Universitaire des Cézeaux, France; 2 Laboratoire Evolution, Génomes et Spéciation, UMR CNRS 9191, Gif-sur-Yvette, France; 3 Saint Quentin en Yvelines, Université de Versailles, Versailles, France; 4 Centro de Investigação de Montanha (CIMO), Instituto Politécnico de Bragança, Campus de Santa Apolónia, Bragança, Portugal; 5 Centre d’Etudes Biologique de Chizé, UMR CNRS 7372, Université de la Rochelle, Villiers-en-Bois, France; 6 Department of Genetics, Physical Anthropology and Animal Physiology, University of the Basque Country (UPV/EHU), Barrio Sarriena s/n, Leioa (Bizkaia), Spain; Universidade de São paulo, BRAZIL

## Abstract

Sociality has brought many advantages to various hymenoptera species, including their ability of regulating physical factors in their nest (e.g., temperature). Although less studied, humidity is known to be important for egg, larval and pupal development, and also for nectar concentration. Two subspecies of *Apis mellifera* of the M evolutionary lineage were used as models to test the ability of a superorganism (i.e. honeybee colony) to regulate the humidity in its nest (i.e. “hygroregulation hypothesis”) in four conservation centers: two in France (*A*. *m*. *mellifera*) and two in Portugal (*A*. *m*. *iberiensis*). We investigated the ability of both subspecies to regulate the humidity in hives daily, but also during the seasons for one complete year. Our data and statistical analysis demonstrated the capacity of the bees to regulate humidity in their hive, regardless of the day, season or subspecies. Furthermore, the study showed that humidity in beehives is stable even during winter, when brood is absent, and when temperature is known to be less stable in the beehives. These results suggest that humidity is important for honeybees at every life stage, maybe because of the ‘imprint’ of the evolutionary history of this hymenopteran lineage.

## Introduction

Terrestrial insects have high sensitivity to temperature and humidity. Most of them cannot control their body temperature (i.e., ectotherm species) and are, thereby, very dependent on their environment [1–3]. Indeed, temperature has an important impact on foraging [1] and it is also crucial for reproduction, larval and pupal development, and the success of the offspring [4]. As superorganisms, eusocial insects have evolved many strategies to maintain their nest temperature stable and controlled [5,6], especially in order to protect the eggs from extreme temperature variations [6]. Nest homeostasis provides not only an incubator for the brood, but also a thermal refuge for individuals to use temperature gradients to regulate their own body temperature [7].

The Eusocial bees use thermoregulation to ensure their survival and health [3,6,8]; for instance, *Apis mellifera* larvae can only survive in an environment with slight temperature fluctuation (i.e., from 32 to 36°C) [9]. The nest thermoregulation strategies of eusocial insects can be passive (e.g., nest orientation, architecture) or active (e.g., clustering, incubation), depending on the species [6]. Those thermoregulation strategies could have an impact on humidity levels [10], a less studied phenomenon although also vital for terrestrial insects [11]. Indeed, environmental humidity is an essential factor to control for the survival of adults and eggs [11].While eusocial insects exhibit a range of humidity preferences in the nest regarding their activity or the presence of brood [11–13], eggs generally require a relative humidity (RH) of above 55% to hatch successfully, with the highest survival varying between 90 and 95% [4,11].

Here, we chose the honeybee, *A*. *mellifera* (Linnaeus 1758) (Hymenoptera, Apidae), as our model organism to assess regulation of humidity in the nest. This insect is present worldwide and has been increasingly studied since the decline of its colonies in many occidental countries [14–16], due to its key role in pollination of many crops and in sustaining the wild biodiversity of ecosystems [14]. While several experiments have been conducted (i) to disentangle the mechanisms that lead to creation and maintenance of microclimate in beehives [9,17,18], and (ii) to determine the impact of parasitism on thermoregulating social behavior [8,19], little is known about regulation of humidity in beehives. The eggs are very sensitive to humidity fluctuations within the nest [11]: a dry atmosphere can lead to the eggs death, either because the embryos die or because the dry egg envelops become too hard for the larvae to hatch [11]. After hatching, humidity is still essential for the brood and the adults survival [20], and it is considered at least as important as temperature for larval and pupal development [4,21,22].

In this study, we investigated for the first time humidity in beehives of native European honeybee subspecies belonging to the M evolutionary branch [23], *A*. *m*. *mellifera* in France, and *A*. *m iberiensis* in Portugal. Our experimental setup includes daily, seasonal and annual measures of relative humidity during an entire year in the beehives to assess the ability of a superorganism (i.e. honeybee colony) to regulate the humidity in its nest (i.e. “hygroregulation hypothesis”), and to determine if geographic, seasonal or sub-species parameters impact this capacity.

## Materials and methods

### Ethics statement

This field study granted by an europenan programm, BioDIVERSA ERANET, in favour of preservation and protection of biodiverstiy, did not not involve endangered or protected species. For all locations in France and in Portugal, no specific permission was required, as the apiaries were outside of Natural Parks. We only had to comply for Portugal with the general regulations about distance between apiaries, which is 800 m from the closest apiary (Decree Law n.o 203/2005).

### European study sites

The study was conducted in four conservation centers created in France and Portugal to preserve the two native M-lineage subspecies. The conservation apiaries were deployed in private lands after obtaining permission from the owners, and to conduct experiments by them on their sites. These conservation centers have an approximate size of 350km^2^. The French conservation centers are located at Rochefort (48°35'47"N; 1°57'57"E) and Pontaumur (45°51’51”N; 2°40’24”E), in the regions of “Ile-de-France” and “Auvergne-Rhône-Alpes”, respectively. The landscape in Rochefort corresponds to that of “plain beekeeping”, and the landscape in Pontaumur corresponds to that of “semi-mountain beekeeping”. The Portuguese conservation centers are located at two latitudinal extremes in Gimonde (41°48’31”N; 6°42’41”W) and Zavial (37°03’14”N; 8°52’40”W), in the regions of “Trás-os-Montes” and “Algarve”, respectively. The landscape in Gimonde corresponds to that of “semi-mountain beekeeping”, and the landscape in Zavial corresponds to that of “plain beekeeping”.

### Experimental design to monitor the relative humidity in hives

In each conservation center, six healthy beehives from the sanctuary zone were randomly chosen to monitor the relative humidity (RH) in honeybee hives. RH was measured by three thermo-hygro button data loggers (also named iButtons). The iButtons were placed in the nest of each of the six monitored colonies, at the two outermost frames (iButton A and C) and at the central frame (iButton B), each of them hanging between two frames by an iron thread. An external thermo-hygro button was placed in each apiary at approximately 200cm high, near the monitored beehives, to register environmental RH variation. Each iButton was programmed to measure RH hourly with a precision of ±1%. Data from iButtons were collected every four months by a reader (Plug & Track, Progues PLUS) and exported to Excel format using the Thermotrack PC V.7 software. The pluviometry (rainfall) data was daily collected at 2 a.m., 8 a.m., 2 p.m. and 8 p.m. by a weather station (Micro El d.o.o., Zagreb) placed in each sanctuary.

### Statistical analysis

To test the “hygroregulation hypothesis”, at daily scale, first, RH (%) measured for each hive was plotted to give an overview of the humidity variation within the hives. Then, the days with the most similar external RH daily profile between the four geographic locations and for two contrasting seasons in beekeeping: summer (high colony activity) and winter (low colony activity) were selected using a similarity index (S) for days without rain in the four conservatories. A similarity matrix S = 1–D, where S denotes similarity and D denotes distance (D (x,y) = (∑_i_ (x_i_—y_i_)^2^)^½^), with x_i_ and y_i_ being the pairs of conservatories to compare, was calculated using Statistica 8.0 software (Stat Soft Inc., Arizona, USA). This index allows us to select one day in winter and one day in summer to compare in-hive RH and external RH in each conservatory.

Then, both days were divided into two parts: the part of the day with gradual decrease in external RH (i.e., from maximum RH, at 6 a.m., to minimum RH, at 5 p.m.), and the part of the day with a gradual increase of external RH (i.e., from the minimum RH of the day, at 6 p.m., until next day’s morning, when RH was maximum, at 5 a.m.). For each selected day, linear regression was calculated using in-hive from iButton B and external data, for each part of the day, to test the daily “hygroregulation hypothesis” (i.e. weak correlation (-0.6 ≤ r ≤ 0,6) linked with a R^2^ ≤ 0.40 (weak coefficient of determination). An index ((RHi-RHe)/RHe) with RHi being the in-hive data of iButton B and RHe being the external RH, was calculated for each hour of each selected day. A clustering analysis was performed with this index in order to classify the hives from each conservatory in a hierarchical way. This analysis was performed using the PermutMatrix 1.9.4 seriation software (SupAgro, Montpellier, France).

An analysis of RH was conducted at the season level, by considering September to November for autumn, December to February for winter, March to May for spring and June to August for summer. For each season and iButton (A, B, C), a non-parametric test, Mann-Whitney, was used to compare the RH data in-hives to the outside ones. For each season, a Mandel’s statistics analysis (statistical test for extreme values) was done to detect if means and variances of RH measured in hives are homogeneous between the four conservation centers. For each season and iButton (A, B, C), a non-parametric ANOVA (Kruskal-Wallis) followed by a Multiple Comparison test (Steel-Dwass-Critchlow-Fligner) were done to compare between conservation centers the RH measured in-hives.

An analysis of RH was also performed for one complete year, from September 2015 to August 2016. In-hive data from each iButton (A, B and C) were put together for each conservation center in order to compare them with the external data, and to study the variability inside and between conservations centers, using XLSTAT Prenium 2018.5. Two data mining analyses, Hierarchical Clustering Analysis (HCA) and Multidimensional Scaling (MDS), were performed to explore the grouping of samples. Then, a non-parametric test, Mann-Whitney, was used to compare for each iButton (A, B and C) the RH data of in-hives to the outside ones during one year. A Mandel’s statistics analysis (statistical test for extreme values) was done to detect if means and variances of RH measured in hives are homogeneous between the four conservation centers. For each iButton (A, B, C), a non-parametric ANOVA (Kruskal-Wallis) followed by a Multiple Comparison test (Steel-Dwass-Critchlow-Fligner) were done to compare the RH measured in-hives between conservation centers.

## Results

### Daily hygroregulation

**Fig 1:**
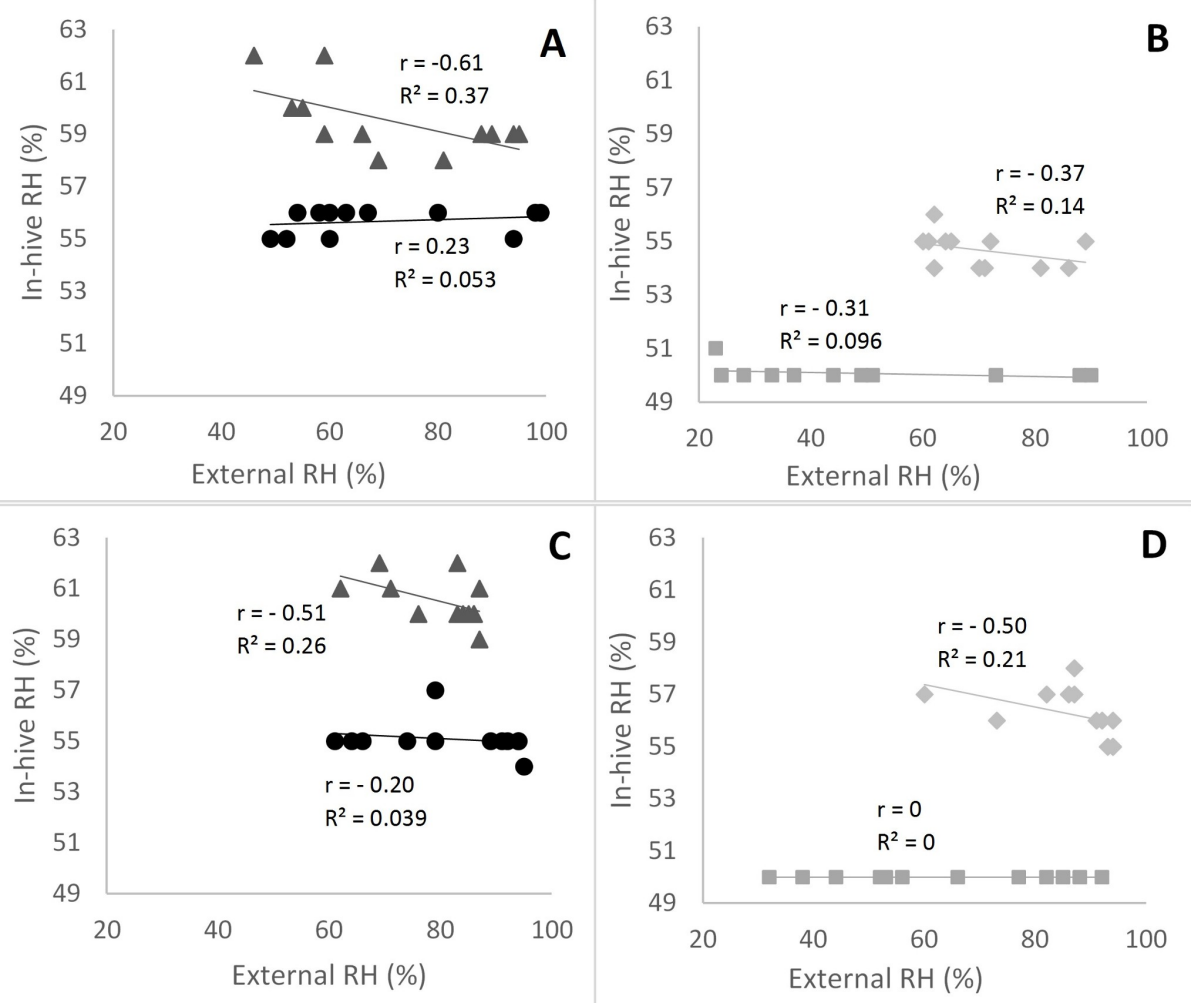
Linear modeling of the relationship between RH observed in the summer in the beehives 1, iButton B (in-hive) with those recorded in their habitat (external) in each conservation center. The modeling was done for one day (July 21, 2016) and separated in two parts: downward RH from 6 a.m. to 5 p.m. and upward RH from 6 p.m. to 5 a.m. the next day. Linear modeling for *A*. *m*. *mellifera* is represented in A (downward RH) and C (upward RH) for Pontaumur (▲) and Rochefort (●). Linear modeling for *A*. *m*. *iberiensis* is represented in B (downward RH) and D (upward RH) for Zavial (♦) and Gimonde (■).

The external RH from the two selected days for the four conservation centers are given in S1 Fig: December 11th, 2015, a winter day without rain or snow, had the most similar external RH profile among the four conservation centers (50% similarity, with RH varying between 23 and 100%), whereas July 21st, 2016, was the most similar not-rainy summer day (40% similarity, with RH varying between 80 and 100%). Regression lines calculated for the central iButton (B) of hive 1 in each conservation center for the summer day are shown in Fig 1 and in S2 Fig for the winter day. These are representative of the regression lines for the remaining five hives and iButtons (i.e., linear equations, r and R^2^), that are shown in S1 (summer) and S2 (winter) Tables.

**Fig 2:**
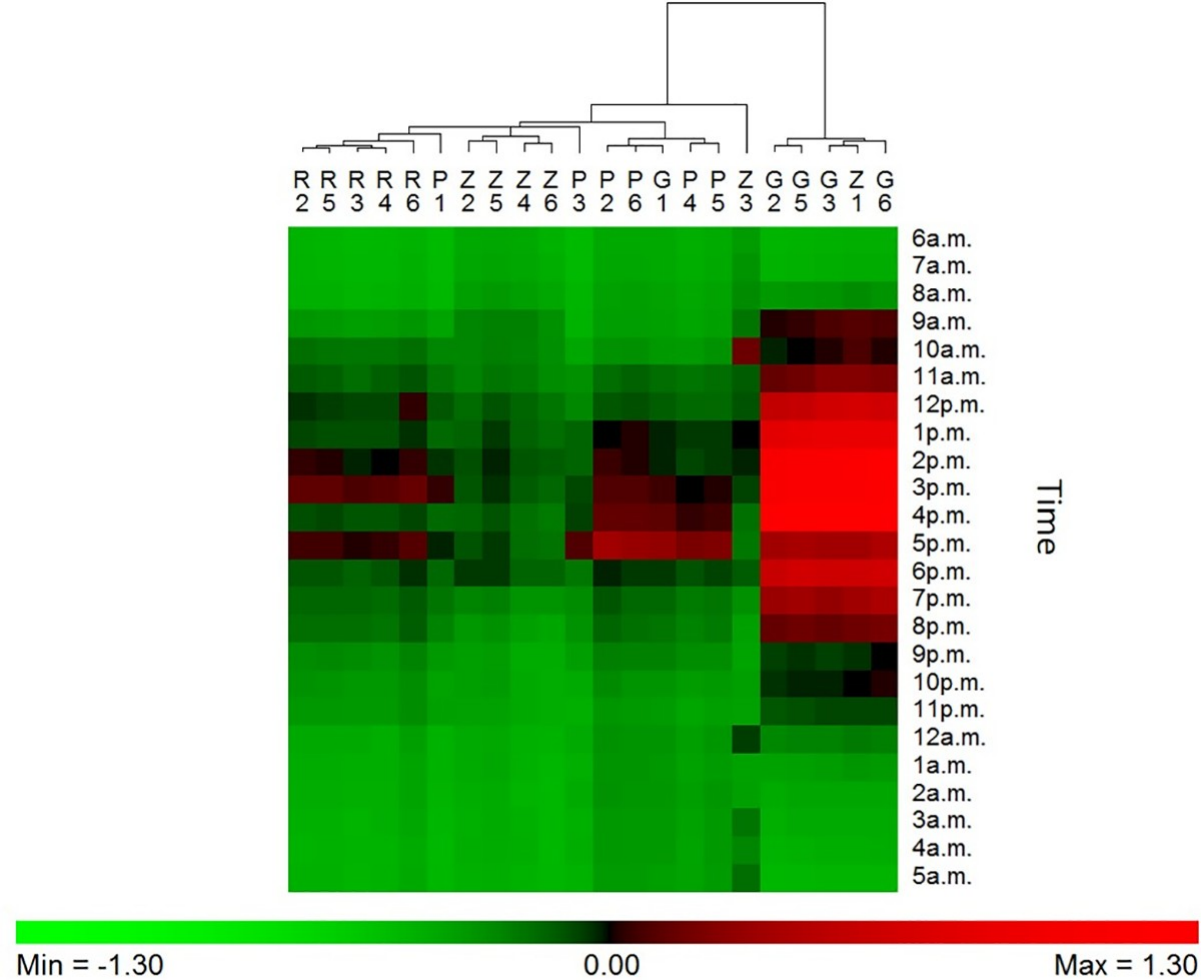
Hygroregulation observed (ratio (RHi-RHe)/RHe) in the central brood frame (iButton B)) of each of the 6 hives during a 24h-period in the summer, from 6 a.m. (July 21, 2016) to 5 a.m. of the next day (July 22, 2016), in the four conservation centers (Rochefort, R; Pontaumur, P; Gimonde, G; and Zavial, Z). Green means that the in-hive RH is lower than the external RH (negative regulation), red means the opposite (positive regulation).

The cluster analysis was only performed for iButton B because it assessed RH in the main part of the brood (i.e. eggs, larvae and pupae) (Fig 2). During summer, the results show globally a positive regulation (in-hive RH > external RH) in the afternoon, especially in Gimonde. In Gimonde, the beehives are mostly maintained with low humidity inside, compared to the other apiaries. During winter, results show a similarity between all conservation centers, since RH is maintained along the whole day at lower level inside than outside the hives (S3 Fig).

### Seasonal and annual hygroregulation

**Fig 3:**
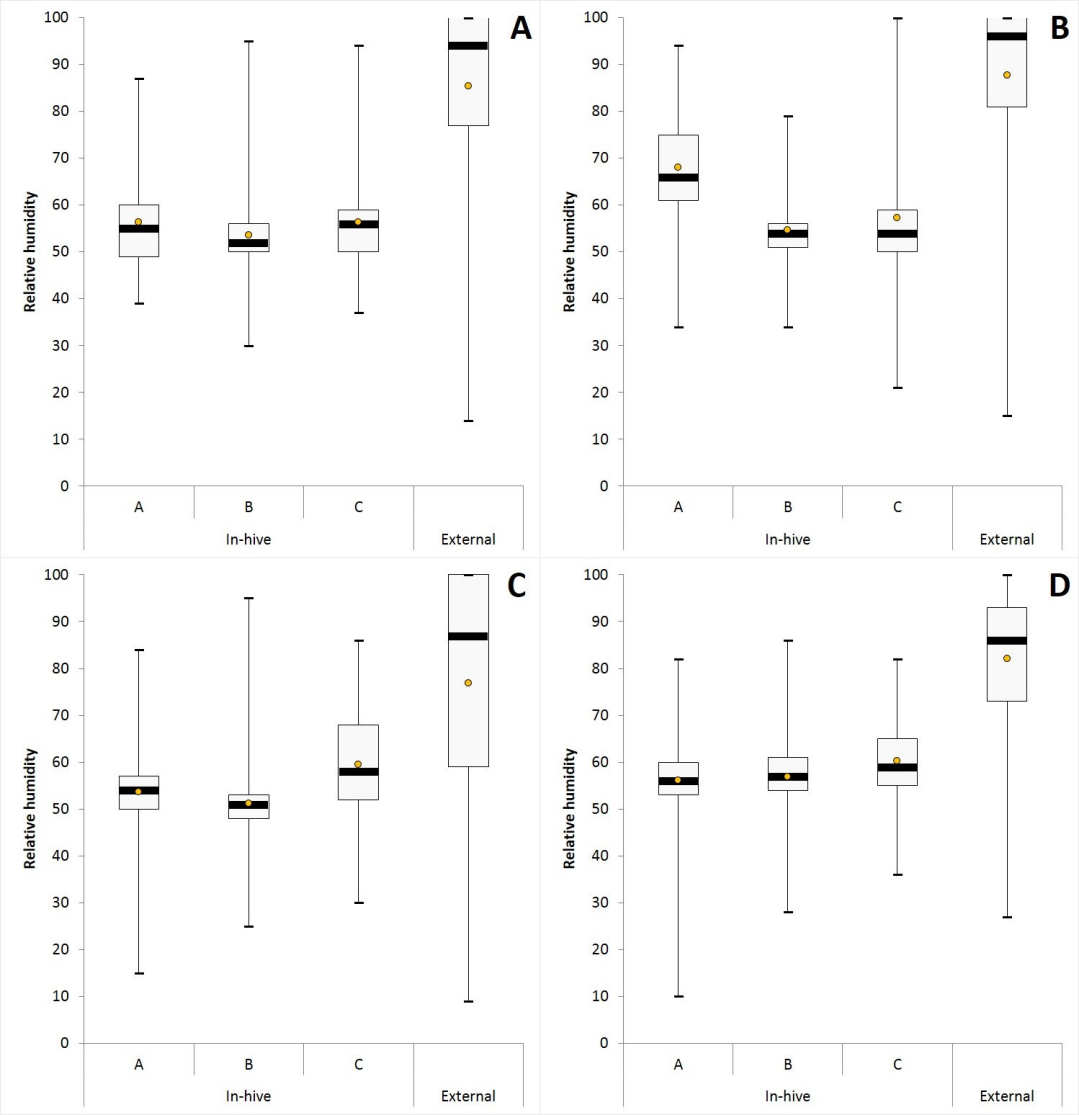
**RH levels over a complete year (September 2015—October 2016) for Pontaumur (A), Rochefort (B), Gimonde (C), and Zavial (D).** Box plots: mean (orange point), median (black stripes) with 1st and 3rd quartiles, maximum and minimum values. For each iButton (i.e. A, B, C) of conservation centers, in-hive RH data were calculated using the six beehives, and external RH was calculated using only the external iButton.

**Fig 4:**
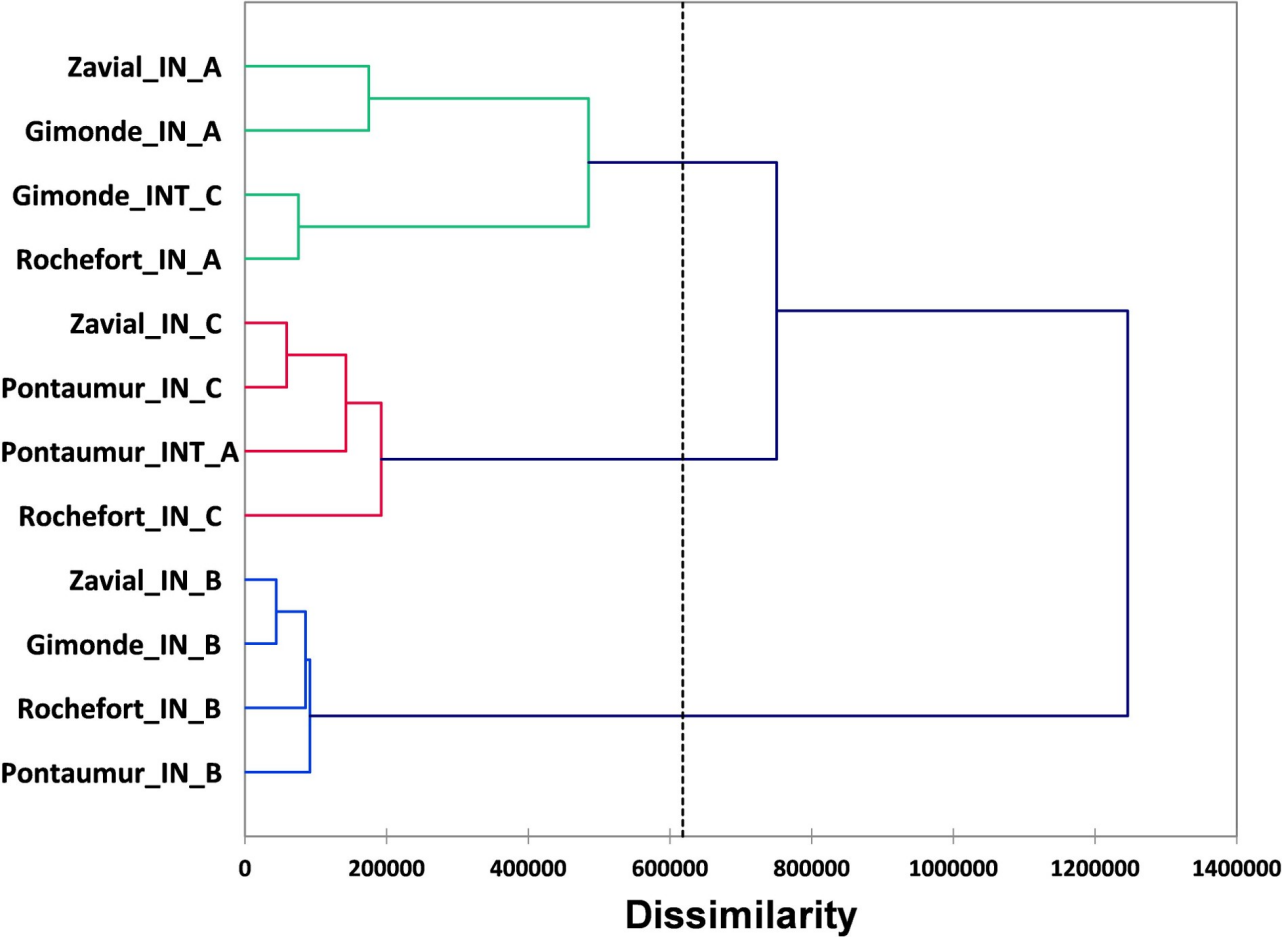
Dendrogram (distance measure: Euclidian; Linkage rule: Ward’s method) resulting of the hierarchical clustering analysis for RH levels measured in hives of the four conservation centers over a complete year for each iButton (A, B, C).

**Fig 5:**
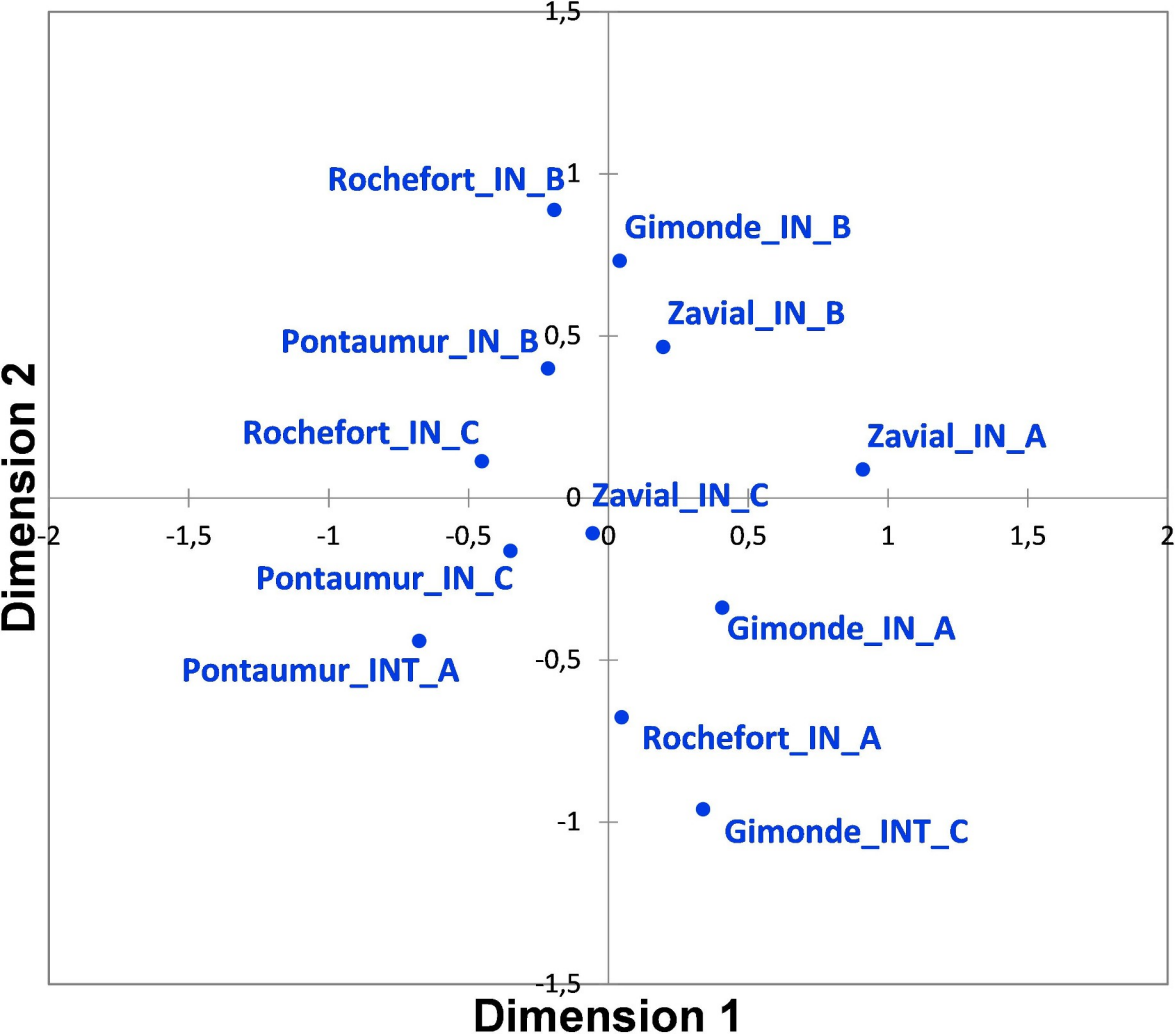
MDS graph resulting of the analysis done with the matrix of dissimilarities (distance measure: Euclidian) for RH levels measured in hives of the four conservation centers over a complete year for each iButton (A, B, C).

**Fig 6:**
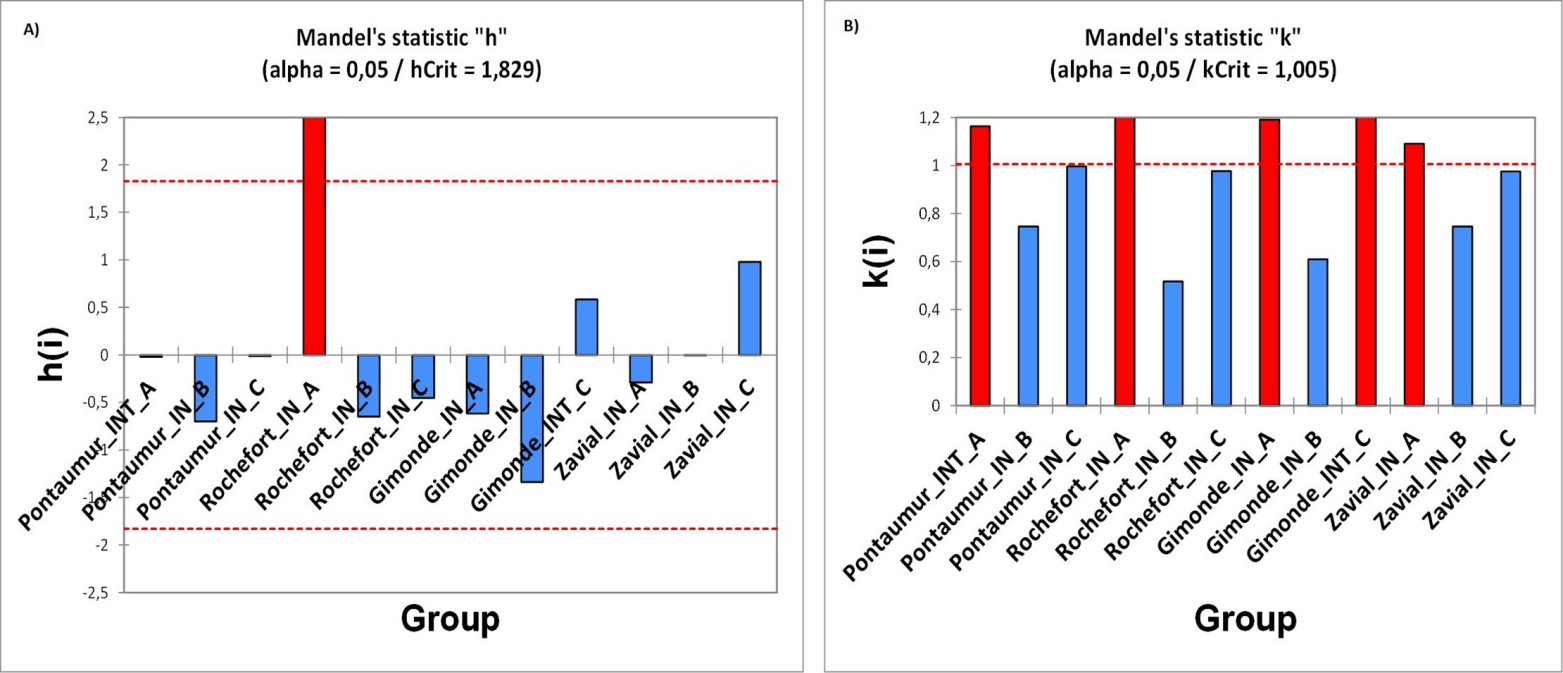
Mandel’s graphs showing results of statistical analysis on homogeneity of means (A) and variances (B) of RH measured in hives of the four conservation centers over a complete year for each iButton (A, B, C).

For RH measured each hour over a complete year in hives of conservation centers, the average, median with 1st and 3rd quartile, the range (maximum and minimum values) for each iButton (A, B, C) are given in Fig 3 and S3 Table. The data mining done by HCA and MDS analysis show a grouping of RH data obtained over a complete year according to the position of iButtons in hives (i.e. iButton B in the center, iButtons A and C both at extremities), and not according to the location of conservation centers (Figs 4 and 5). For each iButton (A, B and C), the RH data in-hives were significantly different (Mann-Whitney U test, p < 0.05) to the outside ones during one year for the two honeybee subspecies deployed in the four conservation centers (*A*. *m*. *mellifera* in Rochefort and Pontaumur, *A*. *m*. *iberiensis* in Gimonde and Zavial) (Fig 3). Mandel’s statistical analysis shows that all conservation centers and iButtons are homogenous for annual average in-hive RH except for the Rochefort’s iButton A (Fig 6A), and also they are homogenous for annual variance in-hive RH for iButtons in position B and C, except for Gimonde (Fig 6B). The comparison of in-hive RH data collected hourly each day over a year shows that conservation centers are statistically different from each other for each position of iButtons (A, B, C) in hives (Kruskal-Wallis ANNOVA followed by the Steel-Dwass-Critchlow-Fligner test,p < 0.05).

The average, median with 1st and 3rd quartile, the range (maximum and minimum values) for each iButton (A, B, C) are given for RH measured hourly for the four seasons in hives of conservation centers, in S4 Fig for autumn, S5 Fig for winter, S6 Fig for spring and S7 Fig for summer. The values are given in S4 Table. The seasonal analysis of RH for each iButton (A, B, C) showed significant differences of RH between the in-hive and the external data (Mann-Whitney U test, p < 0.05), for each conservation center in each season (S4, S5, S6 and S7 Figs). Mandel’s statistical analysis shows that all conservation centers and iButtons are homogenous on each season for the seasonal average in-hive RH, except for the Rochefort’s iButton A (S8A (autumn), S9A (winter), S10A (spring) and S11A (summer) Figs). Moreover, for each season, conservation centers are mainly homogenous for seasonal variance in-hive RH for iButtons, except for Rochefort_IN_A, Gimonde_IN_A and Gimonde_C in autumn (S8B Fig); for Pontaumur_IN_A, Rocherfort_IN_C, Gimonde_IN_C, Zavial_IN_A_in winter (S9B Fig); for Pontaumur_IN_A, Pontaumur_IN_C, Rochefort_IN_A, Rochefort_IN_C and Zavial_IN_A in spring (S10B Fig); for Gimonde_IN_A, Gimonde_IN_C and Zavial_IN_C in summer (S11B Fig).

Furthermore, the statistical analysis shows that, for each season and iButtons (A, B, C), the four conservation centers had significantly different in-hive RH (Kruskall Wallis ANOVA (p < 0.05) followed by Multiple Comparisons (Steel-Dwass-Critchlow-Fligner test,p < 0.05)), (i) except in winter, when Pontaumur (iButton C = 59,8 ± 9,4%) was similar to Rochefort (iButton C = 64.0 ± 17.0%) (Steel-Dwass-Critchlow-Fligner test,p = 0,834), when Gimonde (iButton A = 57.3 ± 7.3) was similar to Zavial (iButton A = 54.9 ± 13.2%) (Steel-Dwass-Critchlow-Fligner test,p = 0,386); and (ii) except in spring, when Pontaumur (iButton A = 54.3 ± 3.3%) was similar to Gimonde (iButton A = 54.9 ± 13.2%) ((Steel-Dwass-Critchlow-Fligner test,p = 0,150).

## Discussion

Eusociality brought advantages to many insect species, especially the ability of living in community and, thus, to regulate some parameters of their immediate environment, such as temperature and humidity. While temperature regulation in superorganisms like eusocial insects has been studied extensively [1,5,6,17,24], providing insights about the factors that influence temperature management [1,25,26], such as climate [27] or genetics [24], there is little information on the humidity regulation inside nests and hives. In this study, we tested the “hygroregulation hypothesis” by means of using as model two native honeybee subspecies from the M branch, *A*. *m*. *mellifera* and *A*. *m*. *iberiensis*, and the assessment of the temporal humidity variation inside and outside beehives. Thereby, we sought to better understand the ability of eusocial insects to regulate the humidity of their nest (hive).

Our study has the advantage to present an approach at different scales, namely the day, the season and the year, in geographic regions with contrasting climates and landscapes. Our daily results overall show low or even no correlation between humidity inside and outside the hive for the two honeybee subspecies, in summer (Fig 1, S2 Table) and in winter (S2 Fig, S1 and S2 Tables). Thus, the honeybee populations in the French and in Portuguese conservation centers maintain constant nest humidity despite the extreme variations that take place outside (Fig 2). In addition, our results show that in-hive RH is more stable than external RH, over the year (Fig 3) but also at seasonal level (S4, S5, S6 and S7 Figs). This illustrates the ability of the two honeybee subspecies to regulate their nest humidity regardless the season, even in the heart of the summer or in the winter, when brood is absent [28]. Furthermore, no differences were found over the year between the two honeybee subspecies, since the in-hive RH in Pontaumur (*A*. *m*. *mellifera*) and Gimonde (*A*. *m*. *iberiensis*) are at the same level, and those from Zavial and Rochefort are higher and close to each other, with Rochefort having always a higher RH level. The similar in-hive hygroregulation in Pontaumur and Gimonde on the one hand, and of Zavial and Rochefort on the other hand, could be due to the environment, Pontaumur and Gimonde being in a semi-mountain place, and Rochefort and Zavial being both in a plain landscape. In addition, Mandel's test showed that the in-hive RH regulation is homogeneous for all iButtons except for Rochefort, where iButton A is slightly higher regardless the season (Fig 6, S8, S9, S10 and S11 Figs). This phenomenon is due to all the hives of the Rochefort conservatory, and can be explained in particular by the hives orientation, the iButton A being on the side that is the most exposed to the wind. Besides, the variance analysis (Box plots and extreme values analysis) highlights differences between seasons, the variances being more important in spring in both hive’s extremities (iButtons A and B), regardless the conservatory (S4 Table, S6 and S10 Figs). This phenomenon can be explained by the activity of the bees, which is higher in spring due to the rebuilding of the colony after winter, and the exit of drones and workers during the day that causes big populations variations during the days. Moreover, considering the colony as a superorganism, its expression of hygroregulation can be different according to different factors like environment of subspecies.

It has been shown that temperature is better regulated in beehives in summer, when there is brood in the hive, than in winter, [29]. This is likely due to the important role of temperature in egg development; it is known that at too low (i.e. < 33°C) or too high (i.e. > 36°C) temperature eggs, larvae and pupae die [9,30]. Similarly, maintenance of humidity level is crucial for social insects; it directly affects the proper development of eggs, larvae and pupae [4,11], which die when the ambient environment is too dry [11]. Therefore, a minimum of 55% humidity is required for honeybee eggs to hatch, with a maximum survival rate between 90 and 95% [4]. In addition, Ellis *et al*. (2008) have shown that honeybee workers have a marked preference for approximately 75% humidity in the absence of brood [22]. In this study, we obtained lower humidity levels in the beehives, with seasonal RH in the middle of the beehives (iButton B) ranging from 50 ± 3% (Gimonde, autumn) to 60 ± 6% (Zavial, winter) (S4 Table). Seasons when brood is present in the hive (spring and summer) do not differ significantly from the rest of the year (S4 Fig). However, in those two seasons, RH does not drop below 50%, threshold below which eggs cannot hatch [4].

The fact that humidity is stable and, therefore, regulated in the winter, unlike temperature [17,29] suggests two hypotheses: (i) the humidity is more easily maintained in the hive than the temperature, despite the fact that the two honeybee subspecies must reduce their energy efforts because food reserves are limited [31], and (ii) moisture is a more important factor for adult health than previously suggested in the literature [4,11,21,22], especially for adult bees that keep it constant even in the absence of brood. The first hypothesis involves a link between temperature and humidity. Indeed, honeybee subspecies, such as *A*. *m*. *mellifera* and *A*. *m*. *iberiensis*, mainly use active regulation systems to manage the temperature of their nest [6]. This strategy includes ventilation, which has also an impact on ambient humidity [28,29]. Besides, some of these active ventilation behaviors have been shown to directly impact humidity levels in the beehives [18]. The second hypothesis is based on studies by Buxton, who showed in 1932 that insects only drink very rarely and need a moist environment to avoid desiccation, whether they are in the larval or adult stages [11,22].

It is important to note that the evolutionary history of honeybees goes back several million years [23], whereas their encounter with humans dates back only 15,000 years ago [28], when bees moved from nesting in various natural cavities to beehives. Their ability to maintain stable moisture and temperature within the colony may have facilitated the migration of different subspecies to geographical areas with a climate that often varies greatly with the seasons, or particularly arid countries, such as in many parts of Africa [32].

Currently, global warming is causing significant changes in the environment that organisms have to face [33–37]. Since 1990, the average global surface temperature, the environmental factor with the greatest impact on the biosphere, has increased by around 0.9° C, with a faster rise for the minimum than for the maximum [37]. This global warming contributes to the destruction of several habitats and biological invasions in several ecosystems [36,37]. Insects, however, show strong adaptabilities to new climates, for example by modifying their range [36,38] or their period of activity [39]. However, according to our results, the two honeybee subspecies included in this study require a nest with relatively stable and high humidity levels. Some eusocial hymenoptera living in relatively arid areas have adapted their behavior according to their unavailability of water [2,39]. For those social species, the lack of water due to global warming could lead to a significant change in their geographic distribution, in order to survive in those new and limiting conditions.

Moreover, as our data support previous studies and confirm an importance of moisture for both brood and adults of bee species [4,21,22,40–42], it is conceivable that the development of certain diseases may be manifested by a disturbance of the humidity in the nest: either (i) poor moisture control that would favor the occurrence of opportunistic parasites such as Varroa mites whose ability to reproduce is impacted by moisture in hives [43], or (ii) the presence of parasites and pathogens causing weakening of colonies [44–46], which would induce a decrease in the ability of insects to properly regulate the humidity of their nest. Thus, we suggest that monitoring abiotic factors such as humidity and temperature in honeybee hives could be a strategy for identifying colonies having disturbance in their normal functioning as a eusocial community, and help to find the eventual factors leading to the decline of a honeybee colony.

## Conclusion

Our data and statistical analysis sustain the validation of the “hygroregulation hypothesis”: the ability of a superorganism (i.e. a honeybee colony) to regulate the humidity in its nest, at a day, but also at seasonal and year scales. Thereby, humidity is constant during the year in the beehives, even in winter when temperature is less regulated because of the absence of brood. Furthermore, the slight differences observed between the seasons can be due to the increase of the colonies’ activity during spring. Overall, our results help to better understand how humidity level in nest (hive) is regulated in eusocial insects, and its relative importance all year long.

## Supporting information

S1 Table Linear equation, r and R^2^ for each model of the relationship between RH observed in the summer in the beehives (in-hive) with those recorded outside (external). The empty places correspond to iButtons that were absent from the beehive at this moment. Missing data are due to a breakdown for two iButtons for a couple of days.
(XLSX)

S2 Table Linear equation, r and R^2^ for each model of the relationship between RH observed in the winter in the beehives (in-hive) with those recorded outside (external). The empty places correspond to iButtons that were absent from the beehive at this moment. Missing data are due to a breakdown for two iButtons for a couple of days.
(XLSX)

S3 Table Mean, median with 1st and 3rd quartiles, maximum and minimum values of RH levels over a complete year (September 2015 –October 2016) for each conservatory. For each iButton (i.e. A, B, C) of conservation centers, in-hive RH data were calculated using the six beehives, and external RH was calculated using only the external iButton.
(XLSX)

S4 Table Mean, median with 1st and 3rd quartiles, maximum and minimum values of RH levels for each season over a complete year (September 2015 –October 2016). For each iButton (i.e. A, B, C) of conservation centers, in-hive RH data were calculated using the six beehives, and external RH was calculated using only the external iButton.
(XLSX)

S1 Fig Relative humidity (RH) measured by the external iButton in the four conservation centers: Pontaumur (▲), Rochefort (●), Zavial (♦) and Gimonde (■), (A) in summer (July 21, 2016) and (B) in winter (December 11, 2015). These two dates were chosen because they have the most similar external RH among the four conservatories in the two seasons, and because it did not rain in any of the four conservation centers. The data were taken from 6 a.m. to 5 a.m. the next day for both dates.
(TIF)

S2 Fig Linear modeling of the relationship between RH observed in the winter in the beehives (in-hive) with those recorded in their habitat (external). The modeling was done for one day (December 11, 2015) and separated in two parts: downward RH from 6 a.m. to 5 p.m. and upward RH from 6 p.m. to 5 a.m. the next day. Linear modeling for *A*. *m*. *mellifera* is represented in A (downward RH) and C (upward RH) for Pontaumur (▲) and Rochefort (●). Linear modeling for *A*. *m*. *iberiensis* is represented in B (downward RH) and D (upward RH) for Zavial (♦) and Gimonde (■).
(TIF)

S3 Fig RH regulations observed in winter for each colony (iButton B) regarding to the time of the day: From 6 a.m. to 5 p.m. the next morning. Green means the in-hive RH is lower than the external RH (negative regulation), red means the opposite (positive regulation).
(TIF)

S4 Fig **RH levels during autumn 2015 for Pontaumur (A), Rochefort (B), Gimonde (C), and Zavial (D).** Box plots: mean (orange point), median (black stripes) with 1st and 3rd quartiles, maximum and minimum values. For each iButton (i.e. A, B, C) of conservation centers, in-hive RH data were calculated using the six beehives, and external RH was calculated using only the external iButton for each conservation center.
(TIF)

S5 Fig **RH levels during winter 2016 for Pontaumur (A), Rochefort (B), Gimonde (C), and Zavial (D).** Box plots: mean (orange point), median (black stripes) with 1st and 3rd quartiles, maximum and minimum values. For each iButton (i.e. A, B, C) of conservation centers, in-hive RH data were calculated using the six beehives, and external RH was calculated using only the external iButton for each conservation center.
(TIF)

S6 Fig **RH levels during spring 2016 for Pontaumur (A), Rochefort (B), Gimonde (C), and Zavial (D).** Box plots: mean (orange point), median (black stripes) with 1st and 3rd quartiles, maximum and minimum values. For each iButton (i.e. A, B, C) of conservation centers, in-hive RH data were calculated using the six beehives, and external RH was calculated using only the external iButton for each conservation center.
(TIF)

S7 Fig **RH levels during summer 2016 for Pontaumur (A), Rochefort (B), Gimonde (C), and Zavial (D).** Box plots: mean (orange point), median (black stripes) with 1st and 3rd quartiles, maximum and minimum values. For each iButton (i.e. A, B, C) of conservation centers, in-hive RH data were calculated using the six beehives, and external RH was calculated using only the external iButton for each conservation center.
(TIF)

S8 Fig Mandel’s graphs showing results of statistical analysis on homogeneity of means (A) and variances (B) of RH measured in hives of the four conservation centers for each iButton (A, B, C) during autumn 2015.
(TIF)

S9 Fig Mandel’s graphs showing results of statistical analysis on homogeneity of means (A) and variances (B) of RH measured in hives of the four conservation centers for each iButton (A, B, C) during winter 2016.
(TIF)

S10 Fig Mandel’s graphs showing results of statistical analysis on homogeneity of means (A) and variances (B) of RH measured in hives of the four conservation centers for each iButton (A, B, C) during spring 2016.
(TIF)

S11 Fig Mandel’s graphs showing results of statistical analysis on homogeneity of means (A) and variances (B) of RH measured in hives of the four conservation centers for each iButton (A, B, C) during summer 2016.
(TIF)
