# Supplementary figures and images for: Hygroregulation, a key ability for eusocial insects: Native Western European honeybees as a case study
Source: PLoS One. 2019 Feb 8;14(2):e0200048. doi: 10.1371/journal.pone.0200048 (PMC6368279; doi:10.1371/journal.pone.0200048)

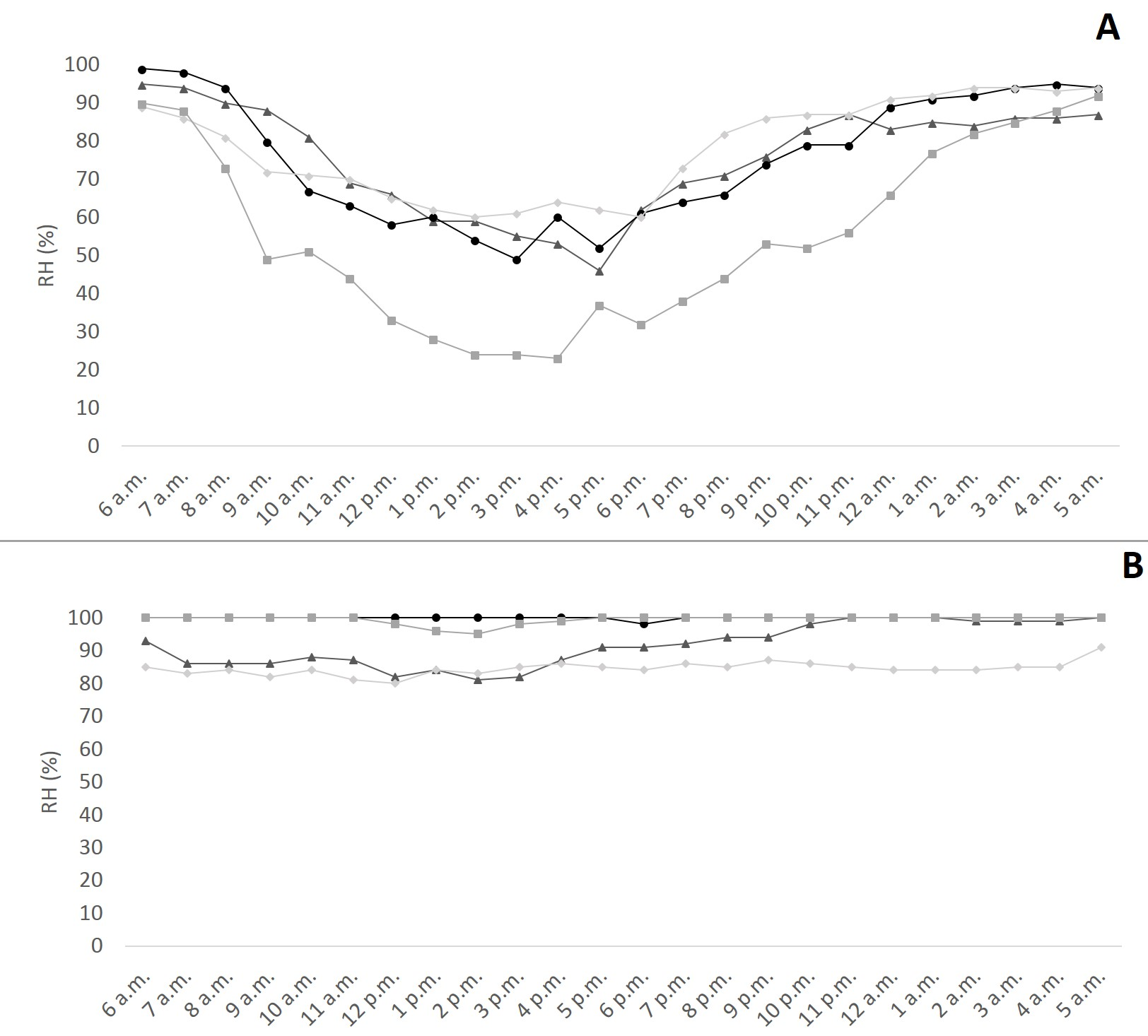

Supplement: S1 Fig — Relative humidity (RH) measured by the external iButton in the four conservation centers: Pontaumur (▲), Rochefort (●), Zavial (♦) and Gimonde (■), (A) in summer (July 21, 2016) and (B) in winter (December 11, 2015). These two dates were chosen because they have the most similar external RH among the four conservatories in the two seasons, and because it did not rain in any of the four conservation centers. The data were taken from 6 a.m. to 5 a.m. the next day for both dates. (TIF) [file pone.0200048.s005.tif]

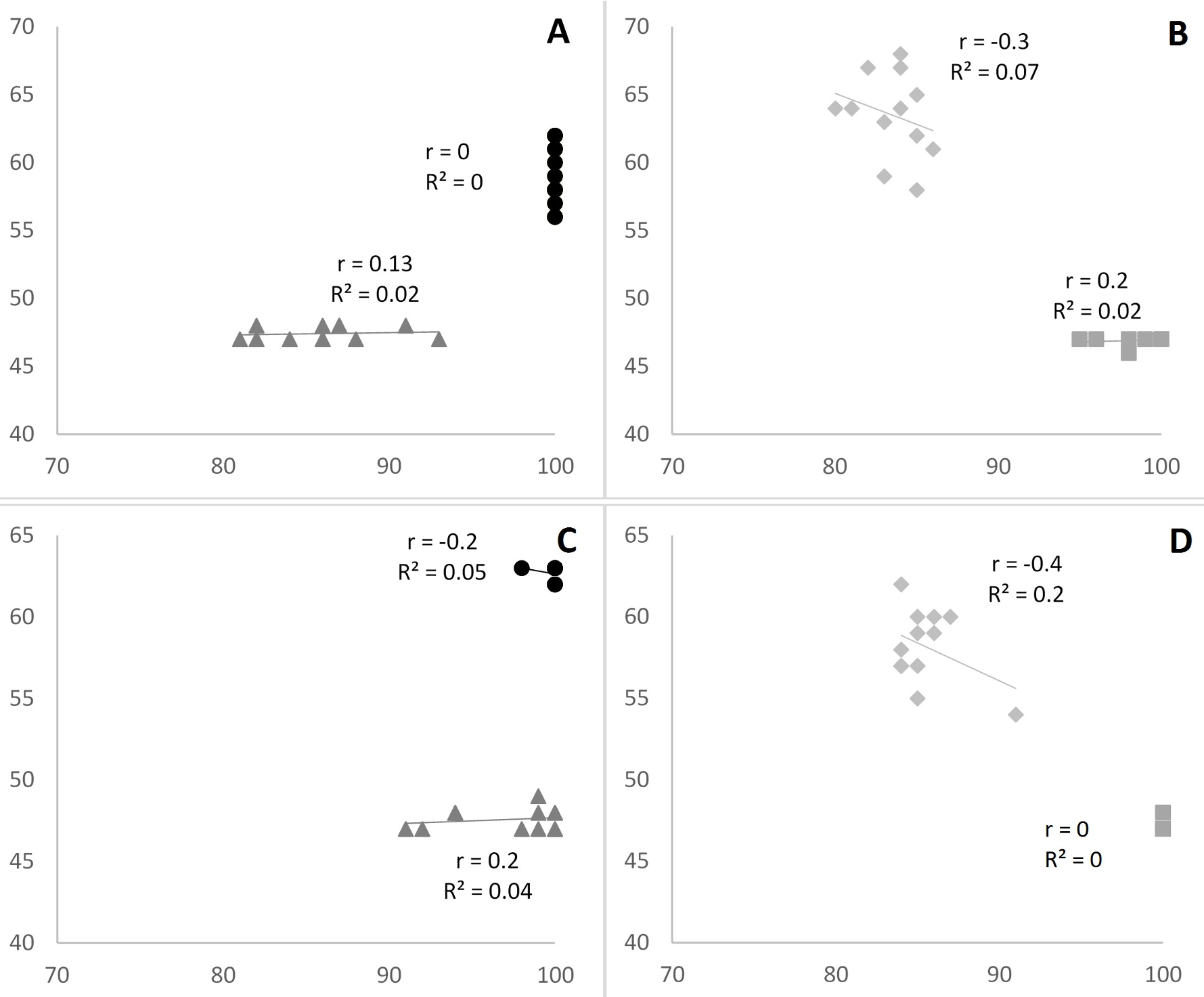

Supplement: S2 Fig — The modeling was done for one day (December 11, 2015) and separated in two parts: downward RH from 6 a.m. to 5 p.m. and upward RH from 6 p.m. to 5 a.m. the next day. Linear modeling for A. m. mellifera is represented in A (downward RH) and C (upward RH) for Pontaumur (▲) and Rochefort (●). Linear modeling for A. m. iberiensis is represented in B (downward RH) and D (upward RH) for Zavial (♦) and Gimonde (■). (TIF) [file pone.0200048.s006.tif]

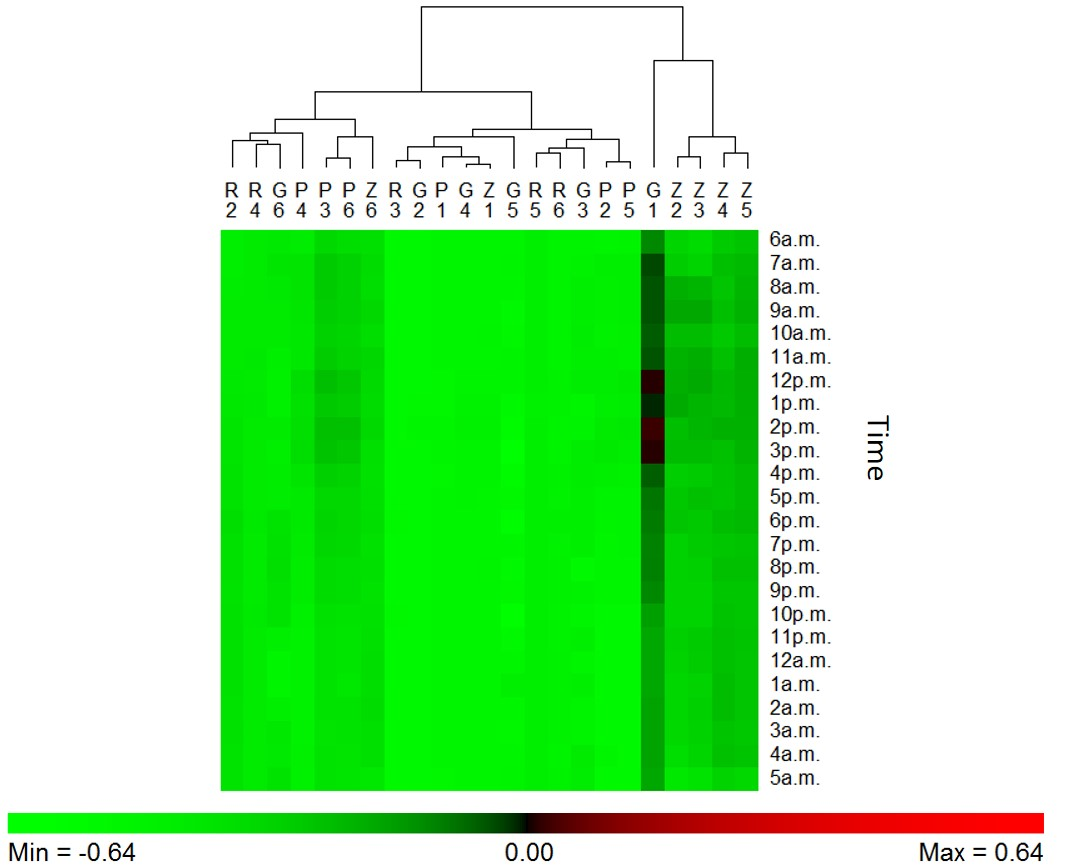

Supplement: S3 Fig — Green means the in-hive RH is lower than the external RH (negative regulation), red means the opposite (positive regulation). (TIF) [file pone.0200048.s007.tif]

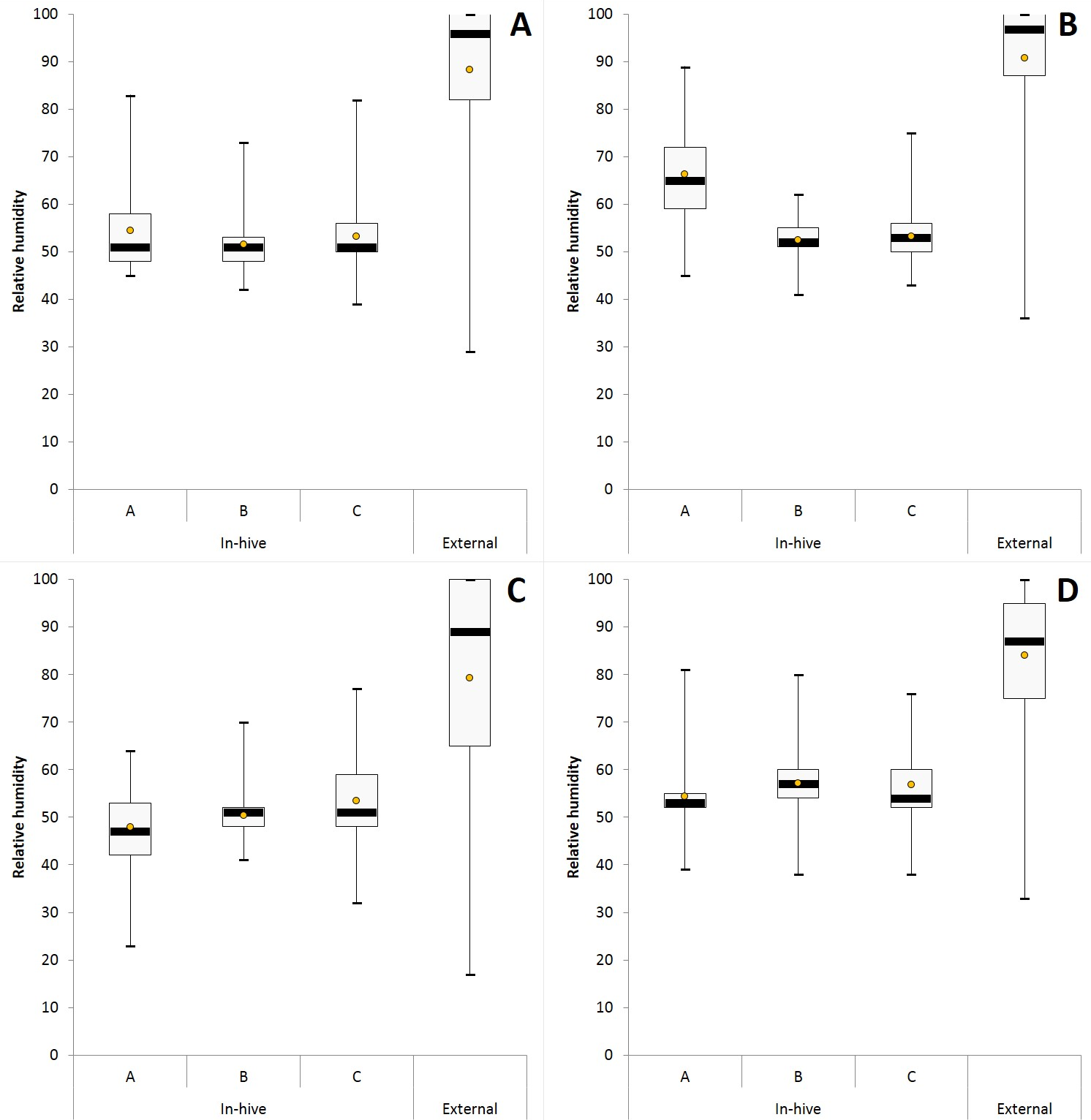

Supplement: S4 Fig — RH levels during autumn 2015 for Pontaumur (A), Rochefort (B), Gimonde (C), and Zavial (D). Box plots: mean (orange point), median (black stripes) with 1st and 3rd quartiles, maximum and minimum values. For each iButton (i.e. A, B, C) of conservation centers, in-hive RH data were calculated using the six beehives, and external RH was calculated using only the external iButton for each conservation center. (TIF) [file pone.0200048.s008.tif]

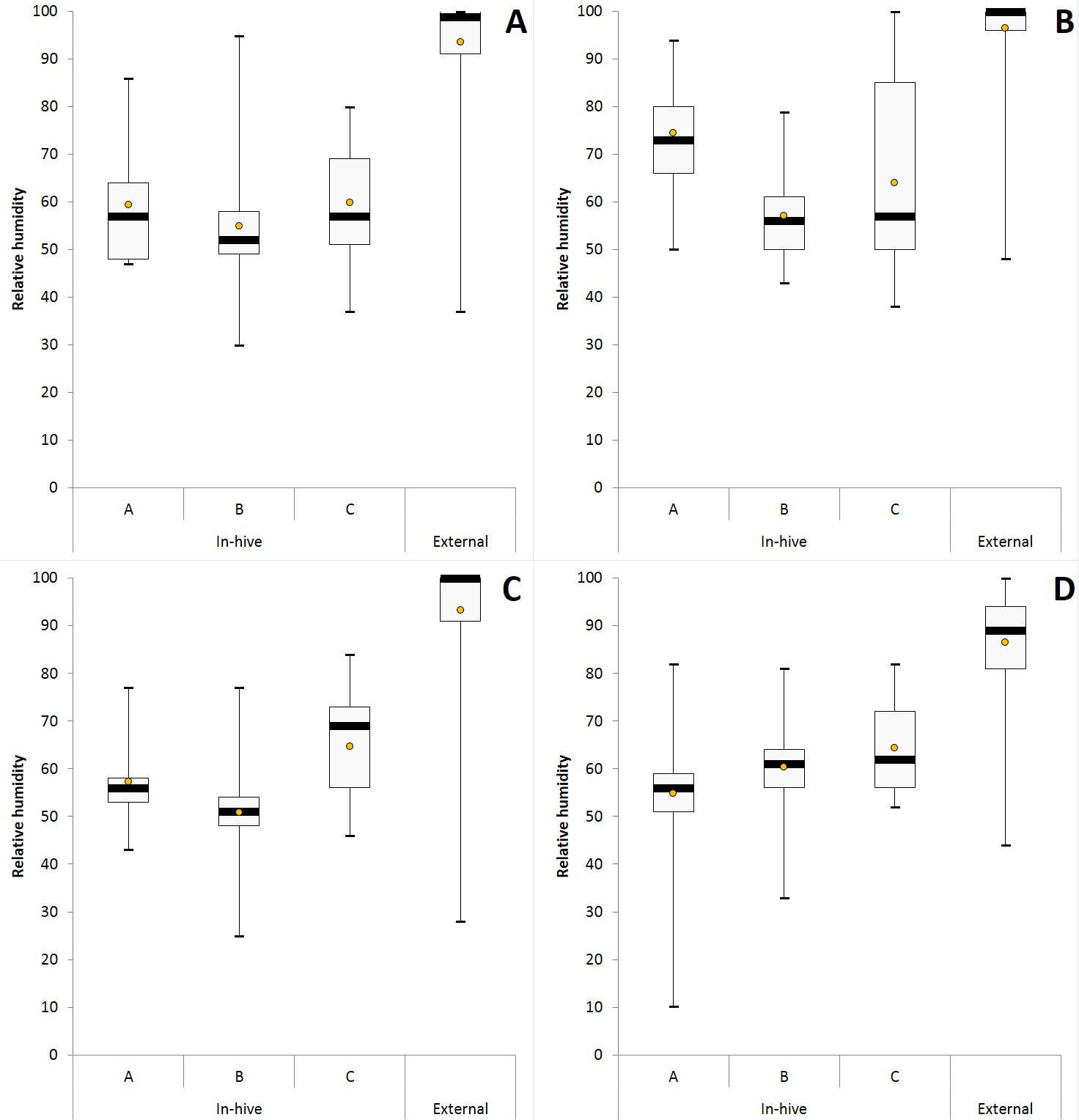

Supplement: S5 Fig — RH levels during winter 2016 for Pontaumur (A), Rochefort (B), Gimonde (C), and Zavial (D). Box plots: mean (orange point), median (black stripes) with 1st and 3rd quartiles, maximum and minimum values. For each iButton (i.e. A, B, C) of conservation centers, in-hive RH data were calculated using the six beehives, and external RH was calculated using only the external iButton for each conservation center. (TIF) [file pone.0200048.s009.tif]

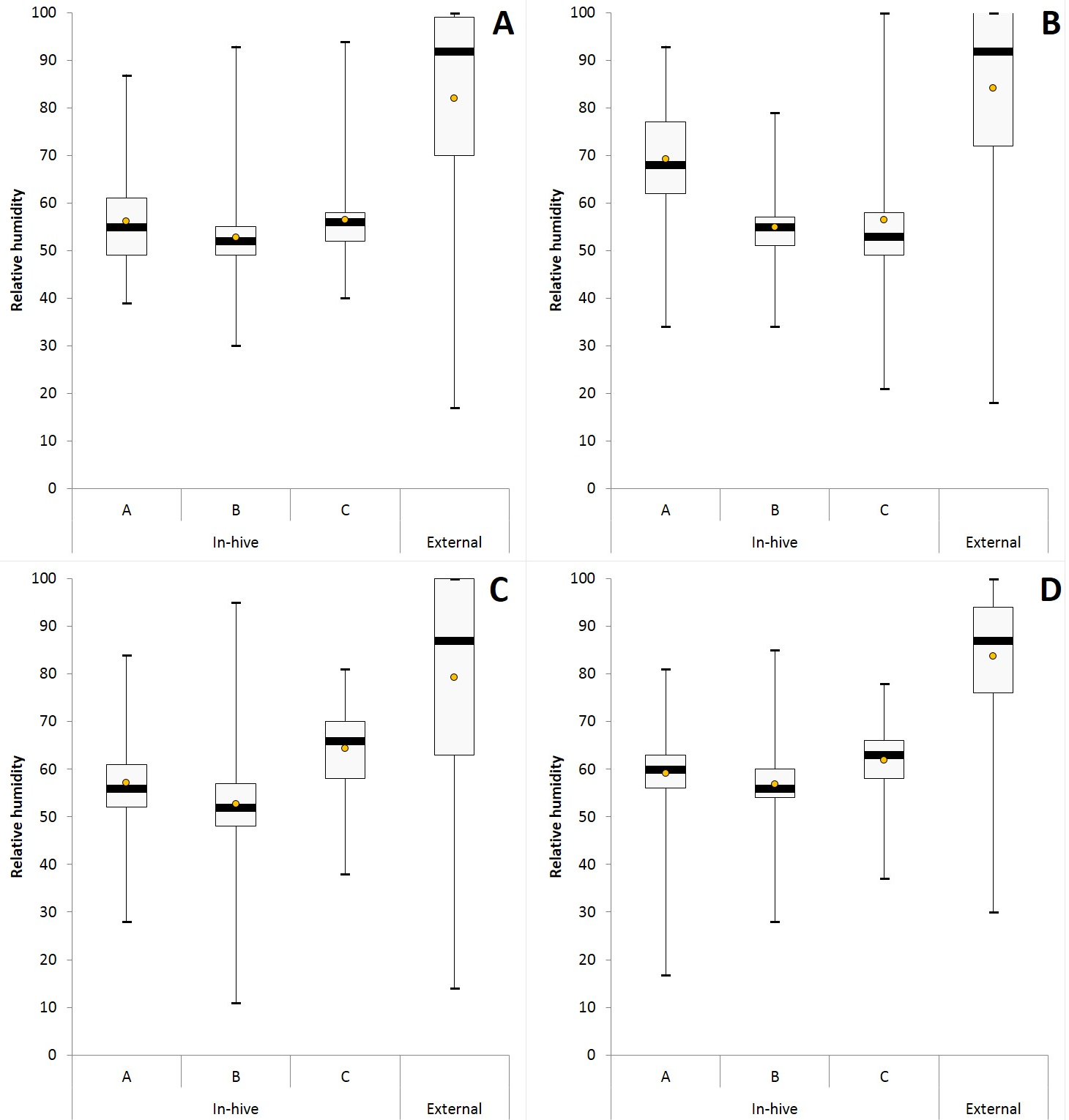

Supplement: S6 Fig — RH levels during spring 2016 for Pontaumur (A), Rochefort (B), Gimonde (C), and Zavial (D). Box plots: mean (orange point), median (black stripes) with 1st and 3rd quartiles, maximum and minimum values. For each iButton (i.e. A, B, C) of conservation centers, in-hive RH data were calculated using the six beehives, and external RH was calculated using only the external iButton for each conservation center. (TIF) [file pone.0200048.s010.tif]

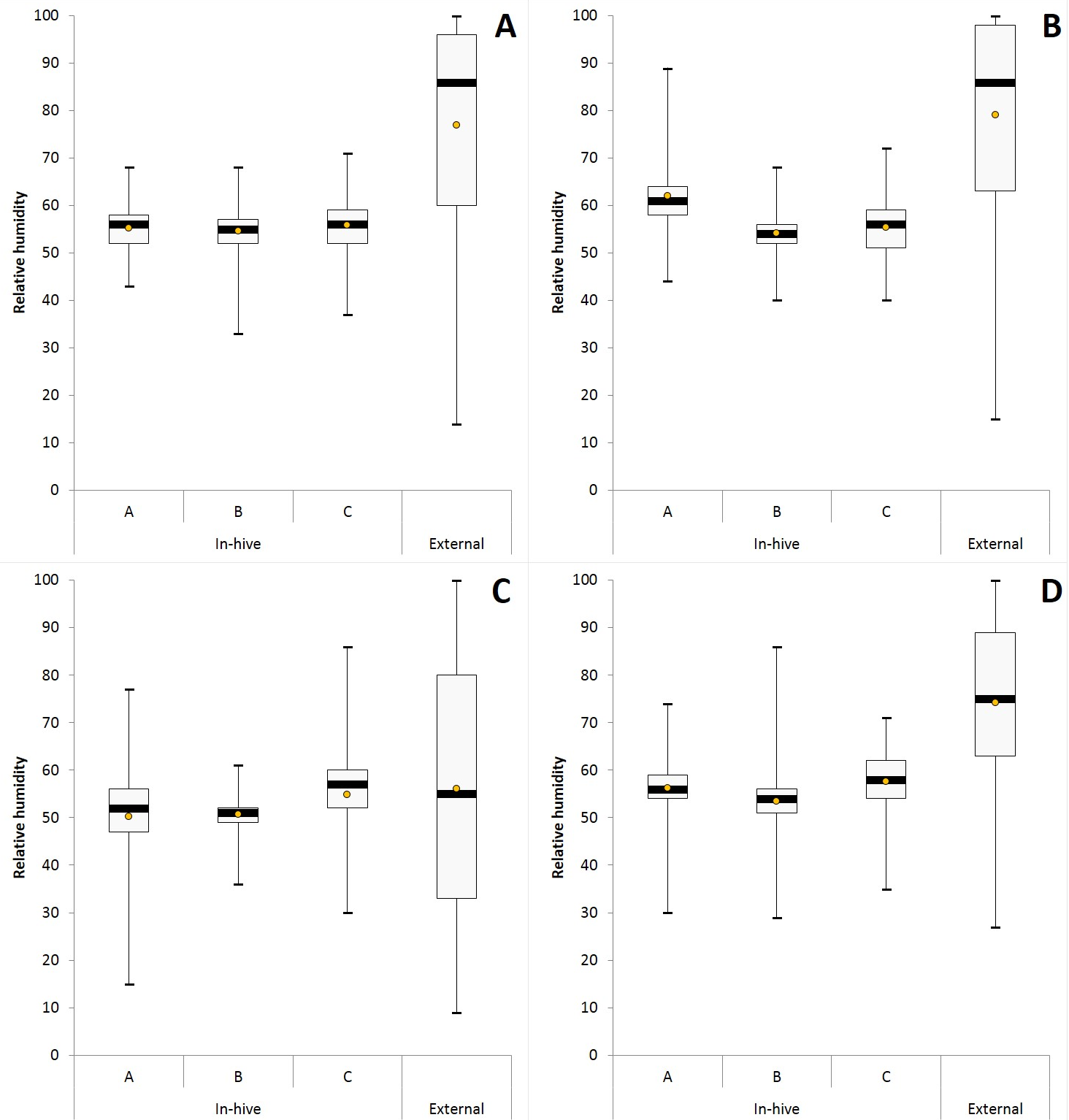

Supplement: S7 Fig — RH levels during summer 2016 for Pontaumur (A), Rochefort (B), Gimonde (C), and Zavial (D). Box plots: mean (orange point), median (black stripes) with 1st and 3rd quartiles, maximum and minimum values. For each iButton (i.e. A, B, C) of conservation centers, in-hive RH data were calculated using the six beehives, and external RH was calculated using only the external iButton for each conservation center. (TIF) [file pone.0200048.s011.tif]

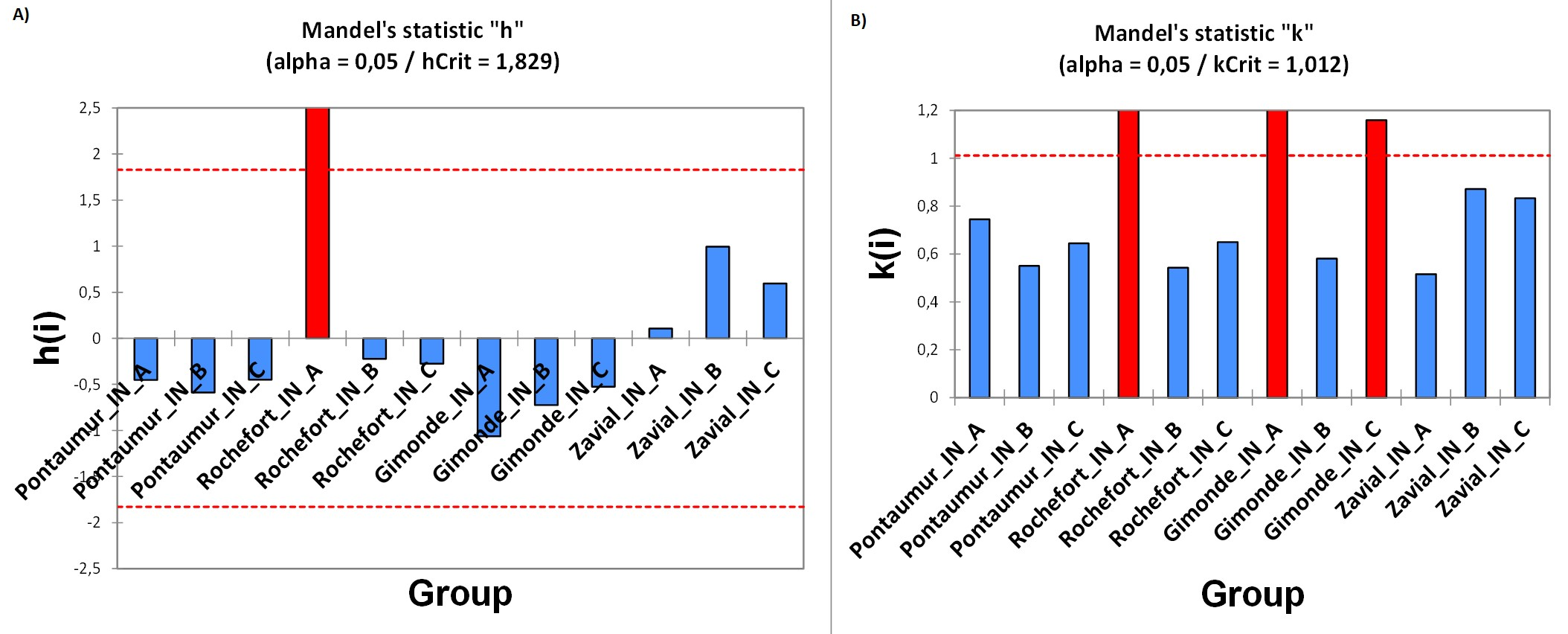

Supplement: S8 Fig — Mandel’s graphs showing results of statistical analysis on homogeneity of means (A) and variances (B) of RH measured in hives of the four conservation centers for each iButton (A, B, C) during autumn 2015. (TIF) [file pone.0200048.s012.tif]

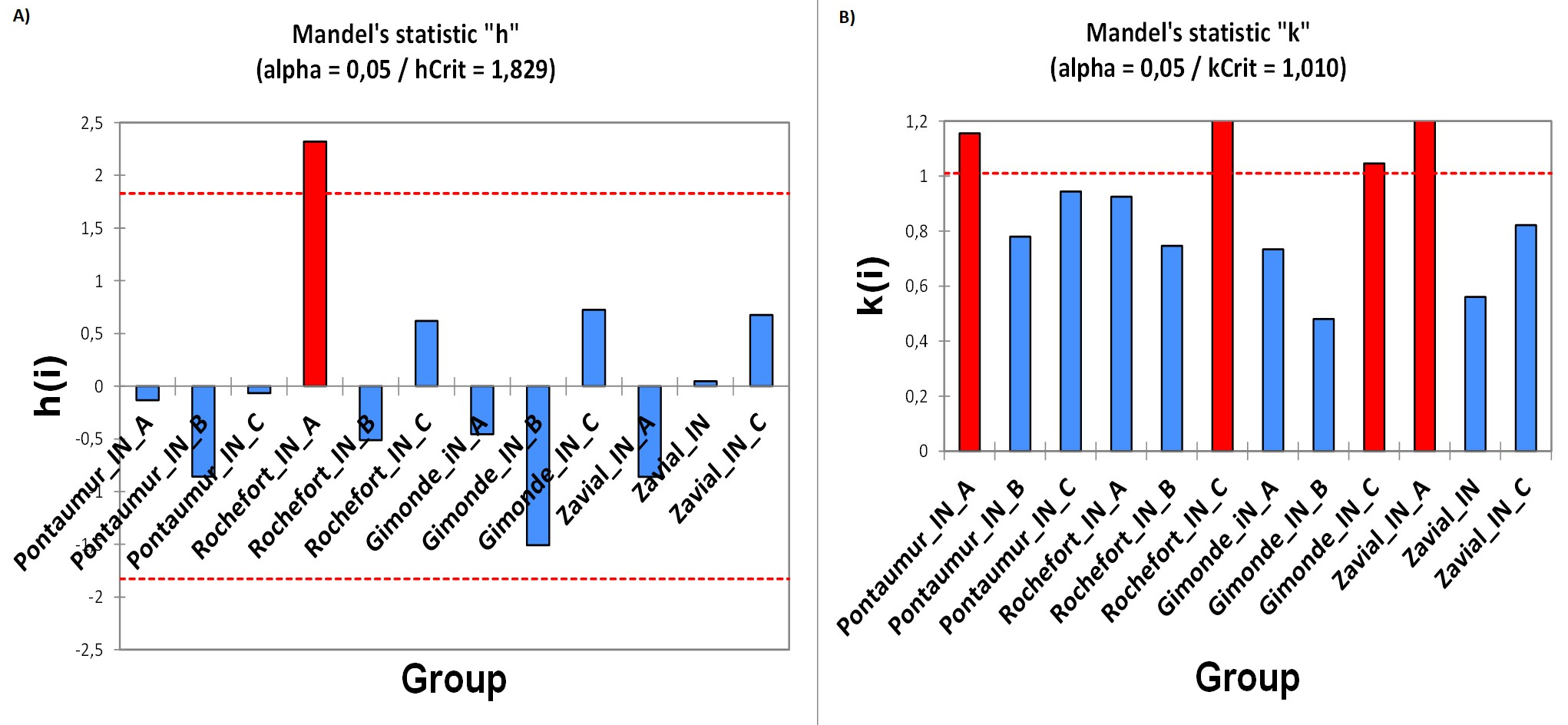

Supplement: S9 Fig — Mandel’s graphs showing results of statistical analysis on homogeneity of means (A) and variances (B) of RH measured in hives of the four conservation centers for each iButton (A, B, C) during winter 2016. (TIF) [file pone.0200048.s013.tif]

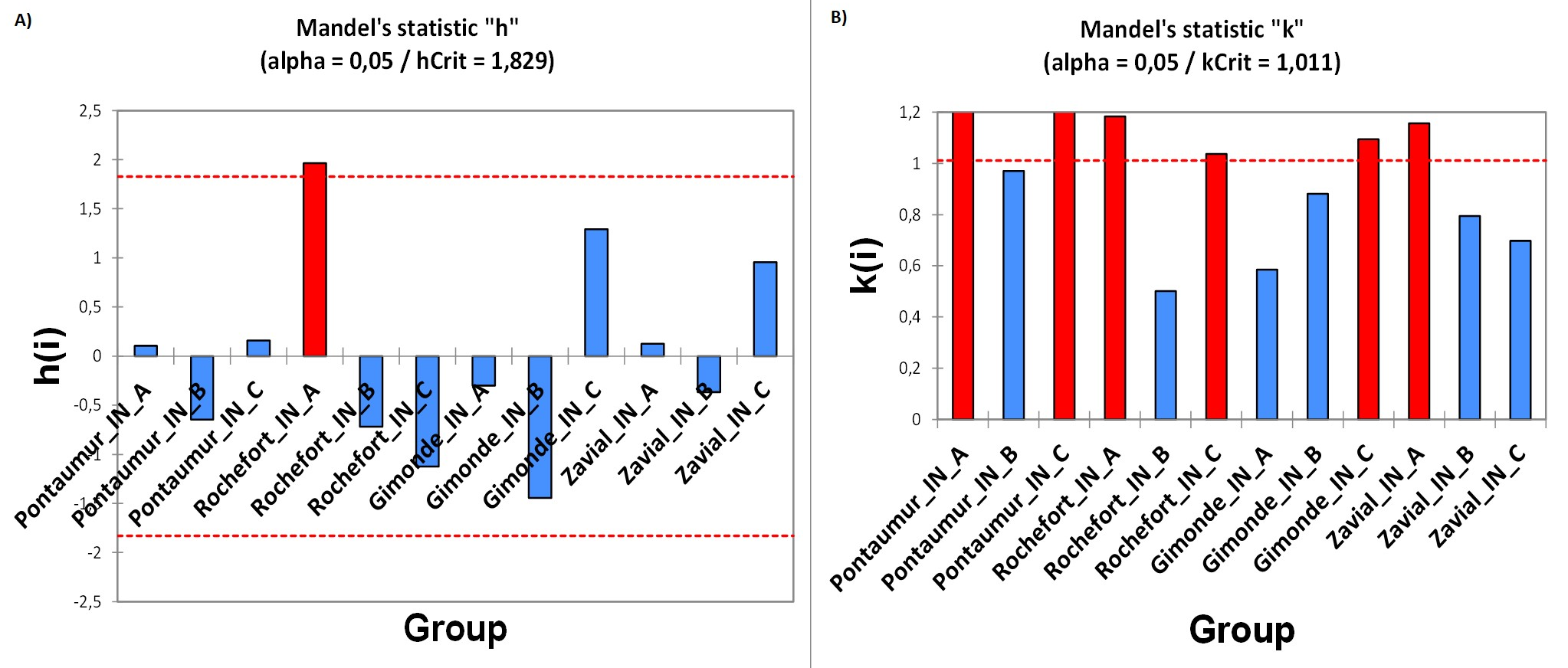

Supplement: S10 Fig — Mandel’s graphs showing results of statistical analysis on homogeneity of means (A) and variances (B) of RH measured in hives of the four conservation centers for each iButton (A, B, C) during spring 2016. (TIF) [file pone.0200048.s014.tif]

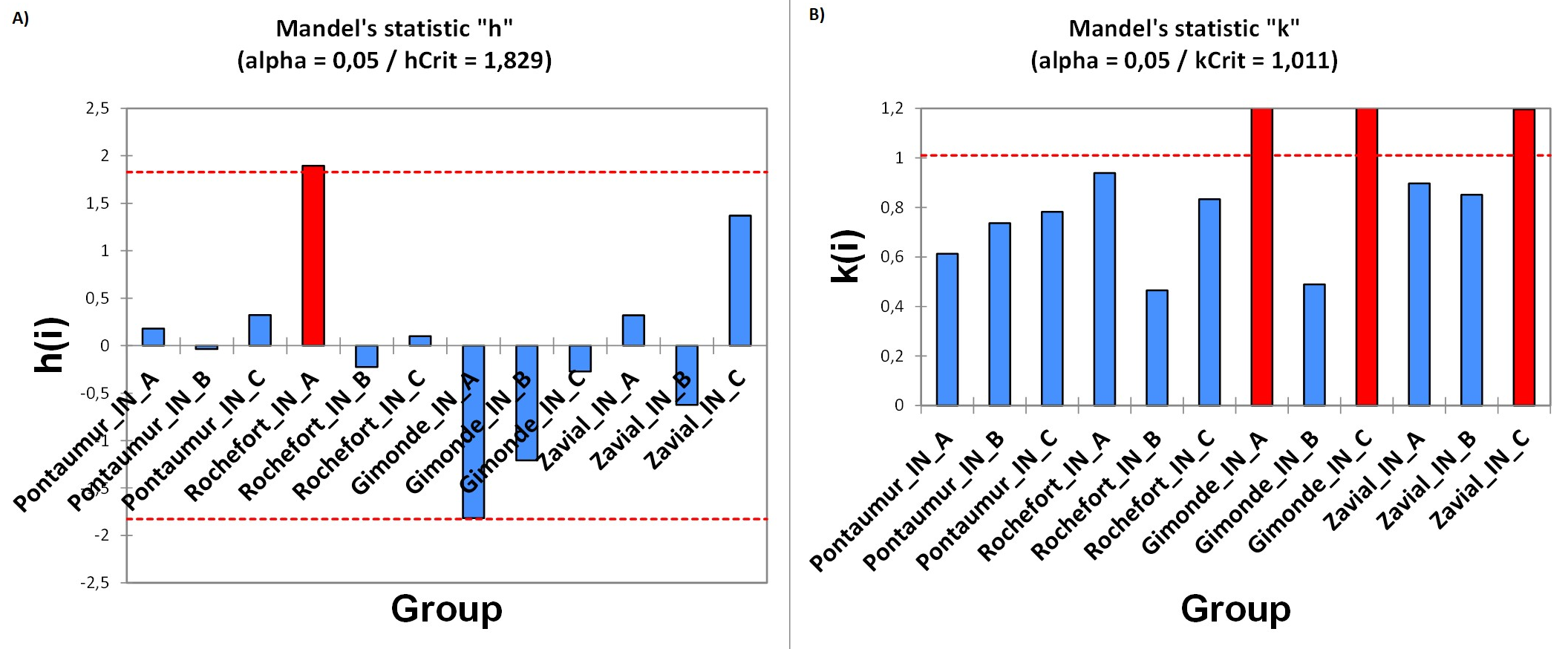

Supplement: S11 Fig — Mandel’s graphs showing results of statistical analysis on homogeneity of means (A) and variances (B) of RH measured in hives of the four conservation centers for each iButton (A, B, C) during summer 2016. (TIF) [file pone.0200048.s015.tif]
